# A motor unit-based model of muscle fatigue

**DOI:** 10.1371/journal.pcbi.1005581

**Published:** 2017-06-02

**Authors:** Jim R. Potvin, Andrew J. Fuglevand

**Affiliations:** 1 Department of Kinesiology, McMaster University, Hamilton, Ontario, Canada; 2 Department of Physiology, College of Medicine, University of Arizona, Tucson, Arizona, United States of America; Johns Hopkins University, UNITED STATES

## Abstract

Muscle fatigue is a temporary decline in the force and power capacity of skeletal muscle resulting from muscle activity. Because control of muscle is realized at the level of the motor unit (MU), it seems important to consider the physiological properties of motor units when attempting to understand and predict muscle fatigue. Therefore, we developed a phenomenological model of motor unit fatigue as a tractable means to predict muscle fatigue for a variety of tasks and to illustrate the individual contractile responses of MUs whose collective action determines the trajectory of changes in muscle force capacity during prolonged activity. An existing MU population model was used to simulate MU firing rates and isometric muscle forces and, to that model, we added fatigue-related changes in MU force, contraction time, and firing rate associated with sustained voluntary contractions. The model accurately estimated endurance times for sustained isometric contractions across a wide range of target levels. In addition, simulations were run for situations that have little experimental precedent to demonstrate the potential utility of the model to predict motor unit fatigue for more complicated, real-world applications. Moreover, the model provided insight into the complex orchestration of MU force contributions during fatigue, that would be unattainable with current experimental approaches.

## Author summary

Skeletal muscle fatigue reduces strength during work and play and profoundly impairs motor function in many neuromuscular disorders. Muscle is composed of groupings of fibres called motor units and these have an extensive range of characteristics from small, weak, and fatigue-resistant to large, strong, and highly fatigable. Our model tracks the fatigue of an entire population of motor units making up a muscle. The model predicted, with good fidelity, the endurance times for a wide range of tasks and provided new insights into the complex orchestration of motor unit contributions to muscle force during fatigue. The model should have wide application in the fields of ergonomics, rehabilitation and exercise to predict and better understand the nature of both motor unit and whole muscle fatigue.

## Introduction

Muscle fatigue is a temporary decline in the force and power capacity of skeletal muscle resulting from muscle activity. Muscle fatigue can adversely affect the lives of workers, athletes, patients, and the elderly—and is a quotidian (and bothersome) presence in the lives of most people. Yet, the basic mechanisms underlying muscle fatigue have not been firmly established. In the periphery, muscle fatigue is thought to arise mainly because of impairments in cross-bridge function and excitation-contraction coupling brought about by accumulation of metabolites and alterations in transmembrane ionic concentrations [1–3]. Centrally, muscle fatigue is manifest as an impairment in activation of the motor neurons that drive muscle fibers. There are a number of factors that likely contribute to this impairment, including diminished output of the higher motor centers that operate on motor neurons, increasing synaptic inhibition directed to motor neurons, and intrinsic adaptations in motor neurons that make them progressively less responsive to synaptic excitation during sustained activity [4–7].

Because control of muscle is realized at the level of the motor unit (a motor neuron and the muscle fibers it innervates), it seems important to consider the physiological properties of motor units (MUs) when attempting to understand and predict muscle fatigue. Indeed, the few hundred MUs that make up a typical mammalian muscle usually possess wide ranges of contractile properties including force capacities, contractile speeds, and fatigabilities. While convention suggests distinct clustering of MUs (i.e. MU types) based on such contractile properties, it is more accurate to represent MU characteristics as residing along broad continua rather than as falling into distinct categories [8]. Control over the diverse population of MUs making up a muscle is enacted in a highly stereotyped way. With few exceptions, MUs appear to be recruited in an orderly sequence, from those that exert the weakest forces toward those that produce the greatest (see [9] for review). Furthermore, there appears to be a tight association between force capacity and fatigability of MUs, such that stronger MUs are more fatigable (ie. fatigue more rapidly) than weaker ones [10–12]. In addition, there is a tendency for weak MUs to have slower twitches (i.e. longer contraction time) than strong motor units [10]. The neural mechanisms underlying the orderly recruitment of MUs—from weakest, slowest and least fatigable, to strongest, fastest and most fatigable—were largely revealed by Henneman and colleagues and is referred to as the size principle [13–15].

Once recruited, individual MUs increase their firing rate with increased synaptic excitation over a relatively narrow range of values before saturating at levels that appear to be inversely related to the MU’s recruitment threshold [16–18]. As such, during a given contraction, MUs within a muscle can possess a wide range of activities, from those not yet recruited to those that have reached their maximal firing rates. If the contraction is sustained, MUs will fatigue at different paces dictated both by their individual firing rates (which can vary over time) and the intrinsic fatigabilities of their innervated muscle fibers. Because of this complexity, it has been difficult to predict the time-course of muscle fatigue, even for relatively simple tasks involving sustained target forces, let alone for tasks in which force levels vary over time and include varying periods of recovery between contractions. Furthermore, when challenged with different tasks, a muscle might eventually accumulate the same level of fatigue (loss in overall muscle force capacity) but do so with very different combinations of fatigue within the individual MUs.

Therefore, our goal was to develop a phenomenological model of motor unit fatigue, not only as a tractable means to predict the mechanical aspects of muscle fatigue across a wide range of tasks, but also to illustrate the varying responses of the individual MUs whose collective action contributes to the trajectory of changes in muscle force capacity during prolonged activity. As such, this model will provide a framework for better understanding physiological mechanisms contributing to the fatigue of individual muscles, and will have applications in ergonomics, rehabilitation, and exercise. While the present paper simulated MU fatigue associated with sustained, isometric contractions only, this work is the first phase of a more comprehensive model to predict MU fatigue *and* recovery for any task demand time-history.

## Results

**Fig 1:**
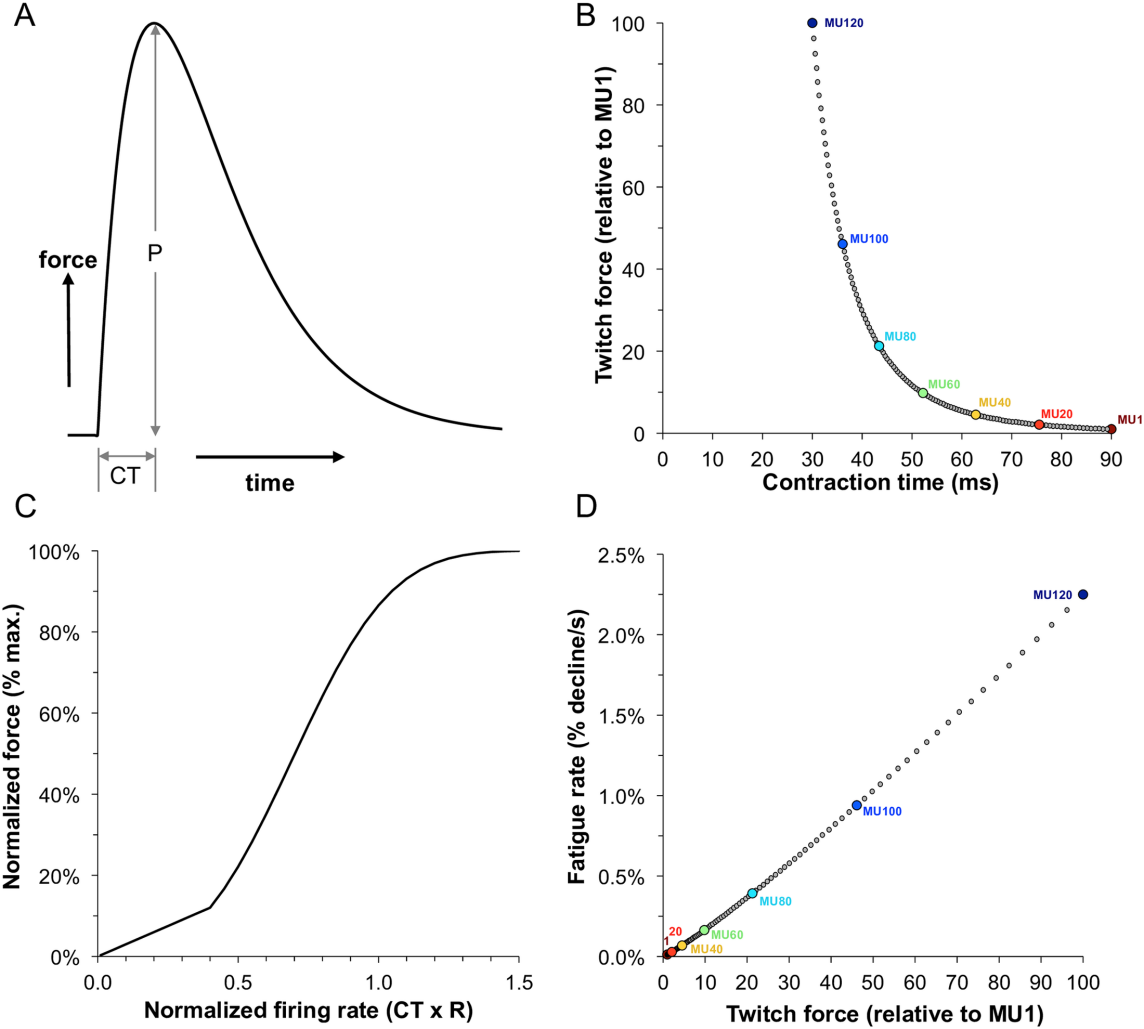
Summary of the motor unit and fatigue model parameters. (A) Example of a twitch response indicating the peak twitch force (P) and contraction time (CT). (B) Inverse relationship between the modeled contraction times and peak twitch forces across the motor units. Values for every 20th MU are shown. (C) Relationship between normalized firing rate and normalized force. This was modeled to be the same for each motor unit. (D) Direct relationship between modeled peak twitch force and fatigability across motor units.

We used an existing MU population model [19] to simulate rested firing rates and isometric forces for a muscle composed of 120 MUs, then added fatigue to individual MUs via central effects on firing rate adaptations and peripheral effects on force capacities and contraction times. The pool had MU characteristics ranging from those that were small, weak and with low fatigability to those that were large, strong and highly fatigable (Fig 1). Motor unit forces were dictated by a force-frequency curve where the input was normalized firing rate (relative to the MU’s contraction time) and the output was normalized force (relative to the MU’s maximum tetanic force). For a detailed description of the model and simulations see 'Material and methods' section.

Here we use the model to address three main questions that would be difficult to address experimentally: (1) how do the force contributions of individual MUs vary during contractions sustained at different target forces? (2) which subpopulations of MUs undergo the greatest degree of fatigue for different types of contractions? and (3) for different tasks but which reach the same overall level of muscle-force loss, are individual MUs fatigued to the same degree across the tasks?

### Submaximal force trials (20% target)

**Fig 2:**
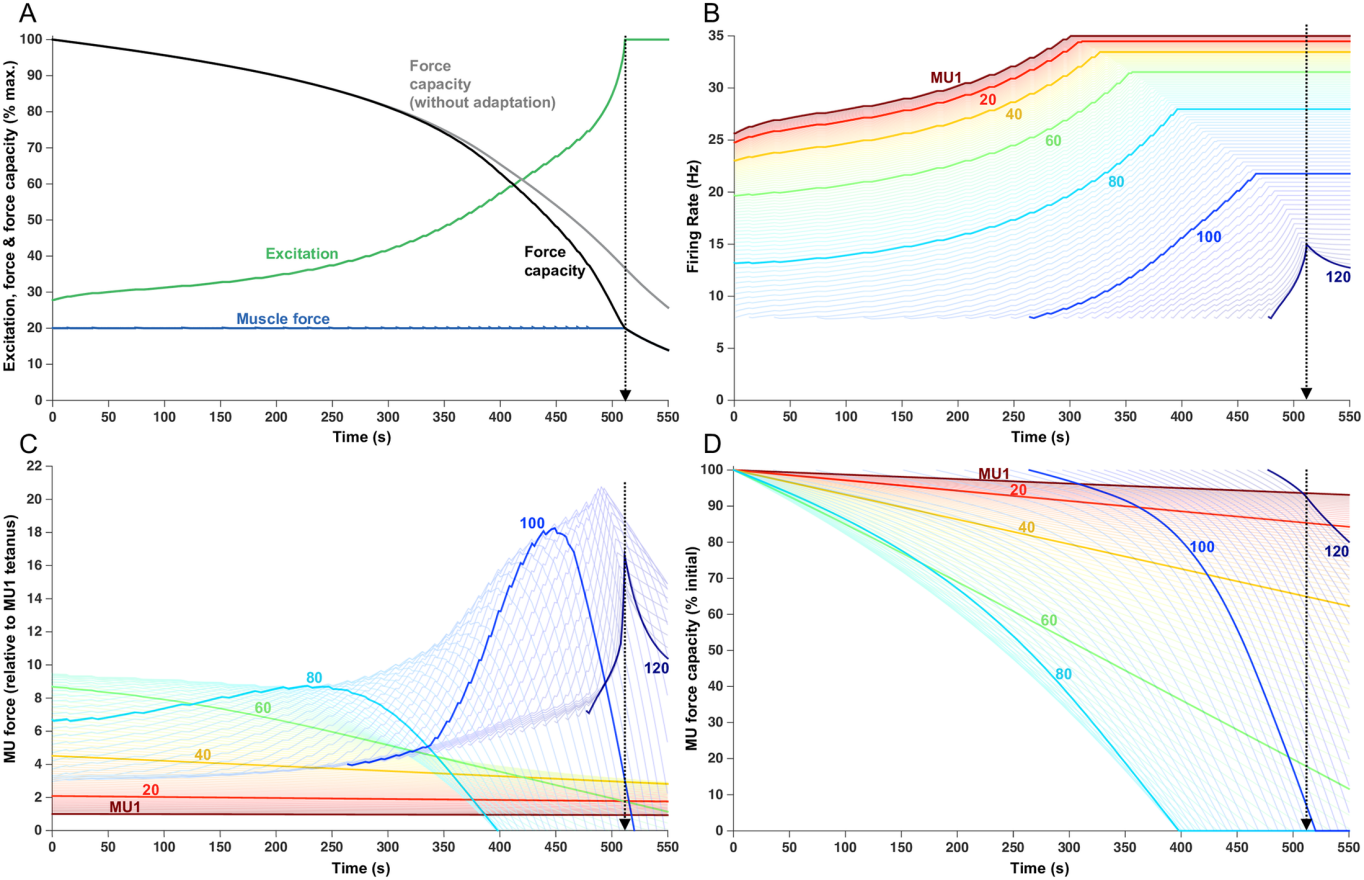
Fatigue model outputs for a sustained 20% MVC force. The endurance time of 511.5 s is indicated with the vertical dotted lines. (A) Increased excitation in response to fatigue. Force capacity is shown with and without firing rate adaptation and the modeled force remains at the target load until the endurance time. (B) Firing rate of each MU, over the course of the trial. Lines begin when the MU was recruited. Each 20th MU is highlighted and labelled, but all 120 MUs are shown as lighter lines. (C) Force contribution of each MU. (D) Relative force capacity of each MU (normalized to its rested capacity).

Fig 2 shows outputs from the model for a simulated sustained 20% force contraction. There was a progressive force-capacity decline over the course of the trial—necessitating an increase in excitation (green trace, Fig 2A) from the initial value of 27.9% maximum voluntary excitation (MVE), to 100% MVE at the endurance time of 511.5 s. The increase in excitation was realized as a gradual increase in firing rates (Fig 2B) up to the assigned maximum rates of those MUs activated from the outset of the contraction (MUs 1–90) and by recruitment and subsequent increase in firing rate of the highest threshold MUs (MUs 91–120). In experimental studies, that have used protocols similar to what was simulated in Fig 2 (i.e. ~20% target force), MU firing rates also tended to increase with time [20–22], not unlike the results of our simulations (Fig 2B). Only a few of the highest threshold MUs, simulated in Fig 2, did not attain their assigned maximal firing rates at the endurance limit because of the countervailing effects of firing rate adaptation, which was assigned to have a more potent effect on high threshold compared to lower threshold MUs.

In the absence of firing rate adaptation (gray line, Fig 2A), muscle force at 511.5 s would have been about 85% higher than that produced in the presence of firing rate adaptation (black line, Fig 2A) and the endurance time would have been extended by about 40 s. Low threshold MUs (e.g. MUs 1–20) initiated their activities close to their maximum firing rates (Fig 2B), which were also close to the normalized firing rates needed for these slow twitch MUs to attain their maximal force (Fig 1C). As such, and because these MUs were fatigue resistant (i.e. assigned low fatigability values, Fig 1D), their force contributions (Fig 2C) remained relatively stable throughout the trial. These low threshold MUs were also the weakest (see Fig 1B) and, as such, their contribution to overall muscle force was modest. Somewhat higher threshold MUs (e.g. MU 40–60) also initiated their firing at relatively high rates (Fig 2B) but these units had slightly higher intrinsic fatigability (Fig 1D) and, consequently, their force decreased gradually throughout the trial.

The highest threshold MUs, recruited from the outset of the trial (e.g. MU80), initially had relatively low firing rates (Fig 2B). In addition, because these units had comparatively brief initial contraction times, their initial normalized firing rates were quite low. For example, the initial firing rate of MU80 was about 13 imp/s (Fig 2B) and it had an initial contraction time of about 43 ms (0.043 s) (Fig 1B). The product of these two values yields a normalized firing rate (Eq 4, Methods) of about 0.58, which placed it quite low on the force-frequency curve (Fig 1C). As firing rate increased, these MUs moved up the steep portion of the force-frequency curve, leading to an initial increase in their force (Fig 2C) which partially compensated for the decreasing force from lower threshold MUs to maintain muscle force at the target level of 20% of maximum (blue trace, Fig 2A). However, because these higher threshold units also had reasonably high fatigability (Fig 1D), their force output eventually started to decline (e.g. at ~ 250 s for MU80) and then declined steeply for the remainder of the trial (Fig 2C).

Motor units recruited later in the trial started firing at the minimum rate and then gradually increased their firing rates as excitation increased (e.g. MU100, Fig 2B). The low starting firing rates, combined with the brief contraction times of high threshold MUs, placed these units initially on the far left, linear portion of the force-frequency curve (Fig 1C). Consequently, as excitation increased, firing rates increased in these units, and force initially increased linearly (Fig 2C, MU 100) then eventually transitioned into the steeper portion of the force-frequency curve and, consequently, force then increased more precipitously (from ~ 340–440 s, Fig 2C). Because these MUs were assigned to have high force capacity (Fig 1B), their force contribution was substantial. These MUs, however, were also the most fatigable and, as such, their force output then decreased steeply.

In addition, because of the imposed ‘onion-skin’ organization (i.e. high threshold MUs assigned the lowest maximum firing rates, see Methods) and the effects of firing rate adaptation, these high threshold MUs increased firing rate over only a relatively modest range. Eventually, the endurance time was reached when no further voluntary increase in any MU's force was possible. Fig 2D shows the force capacity of each MU relative to its initial force. At the endurance limit (~512 s), MU1 had lost only about 5% of its force whereas MUs 20, 40, and 60 had lost ~ 15%, 35%, and 80% of their force, respectively. Interestingly, MUs 66–98 had lost all their force capacity and were essentially exhausted.

### Submaximal force trials (50% target)

**Fig 3:**
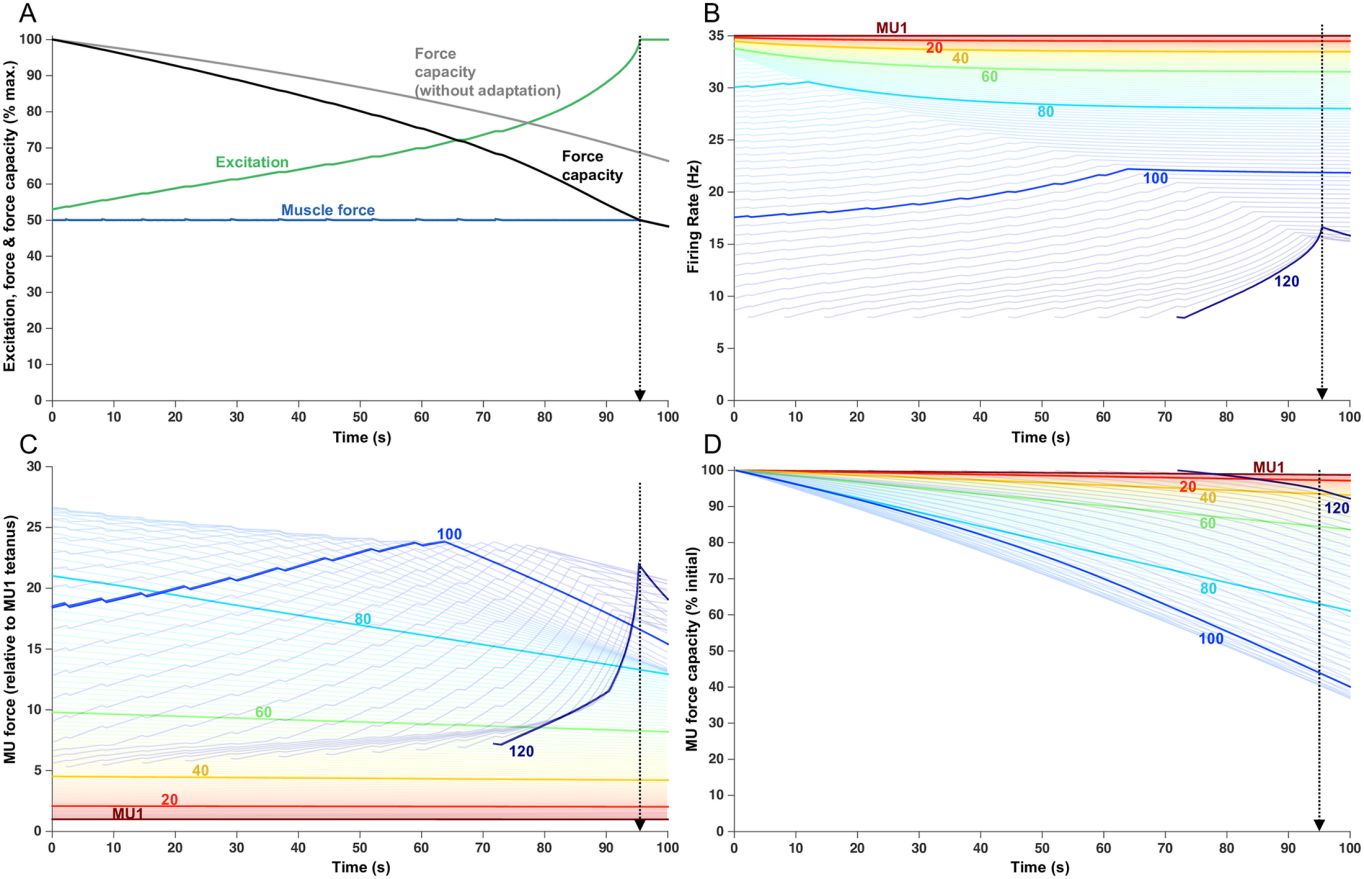
Fatigue model outputs for a sustained 50% MVC load with an endurance time of 95.5 s. (A) Increased excitation in response to fatigue. Force capacity is shown with and without firing rate adaptation and the modeled force remains at the target load until the endurance time. (B) Firing rate of each MU, over the course of the trial. Lines begin when the MU was recruited. Each 20th MU is highlighted and labelled, but all 120 MUs are shown as lighter lines. (C) Force contribution of each MU. (D) Relative force capacity of each MU (normalized to its rested capacity). Note the higher y-axis scale (30) than with the 20% MVC force (22).

Fig 3 shows the simulation of a sustained contraction at 50% of maximum force. Excitation (green trace, Fig 3A) increased over the trial, at a rate substantially greater than for the 20% force trial, to maintain the 50% target force in the face of the progressively declining total muscle force capacity (black trace, Fig 3A). At 95.5 s, muscle force capacity dropped below the target level of 50%, thereby demarking the endurance limit for this trial. If firing rate adaptation was not included in the simulation (gray trace, Fig 3A), muscle force capacity at 95.5 s was well above the target force and the endurance time was extended to ~132 s.

MUs 1–109 were recruited from the outset of the contraction (Fig 3B). Of these, MUs 1–72 initiated their activities already at their assigned maximum firing rates. As a result, the firing rates of these MUs declined over time due to the influence of firing rate adaptation. Higher threshold MUs in this group exhibited greater degrees of firing rate adaptation than lower threshold MUs (e.g. compare MU 60 to MU40, Fig 3B).

MUs recruited at from the start of the contraction, but with firing rates less than their assigned maximum (e.g. MU 80, Fig 3B), initially increased firing rates in response to the escalating excitation. However, the rate of firing rate increase was less than the rate of excitation increase because of the competing effect of firing rate adaptation. MUs with high initial firing rates (e.g. MU 80) eventually reached their maximal rates, after which time their firing rates declined due to adaptation. MUs activated from the outset, but with lower initial rates (e.g. MU 100), gradually increased their firing rates but did not reach their maximal firing rates. This failure to reach maximal firing rates occurred because, over time, the increasing effects of adaptation undercut the effects of increases in excitation. In some cases (e.g. MU 100), a near balance was struck between these two competing influences leading to a leveling-off in firing rate.

As a consequence of the complex interaction between excitation (tending to drive the pool of MUs as a collective) and adaptation (an intrinsic effect that influences the firing rates of individual MUs), an array of firing rate profiles was observed. Indeed, some MUs showed progressive decreases in firing rate (low threshold MUs), some showed increases followed by decreases in firing rate, and others showed mainly progressive increases in firing rate. Furthermore, at any point in time, a range of firing rate responses could be observed. For example, at about mid-way through the contraction (~50 s), some MUs had stable firing rates, some had slowly decreasing firing rates, others had increasing firing rates, and some units were just being recruited. Such disparate firing rate responses across motor units have also been observed in human motor units during fatiguing contractions (e.g. [20,21,23–29]).

Like that for the 20% force contraction, lower threshold MUs (i.e. MU 1–60) showed little drop in force over the duration of the contraction held at 50% force (Fig 3C). These units were assigned low values of fatigability (Fig 1D) and, also, exhibited little firing rate adaptation. As such, their force contributions (although comparatively small) were relatively stable during this simulation. Higher threshold MUs, that were recruited from the outset and with high initial firing rates (e.g. MU 80, Fig 3C), showed a progressive decline in force capacity over the course of the contraction due to both relatively high values of assigned fatigabilities and greater degrees of adaptation, compared to lower threshold MUs. MUs recruited from the outset at low rates (e.g. MU 100), and those units recruited after the onset of the contraction, initially increased their force contribution due to increasing firing rates. Eventually, however, when diminishing intrinsic excitability (associated with firing rate adaptation) matched or exceeded the degree of increasing extrinsic excitation, firing rates leveled off or started to decrease (see Fig 3B). Consequently, force then dropped steeply for these high threshold units that were assigned the highest fatigability values (Fig 1D).

At the endurance limit, lower threshold MUs (e.g. MUs 1–60) still retained at least 90% of their force capacity (Fig 3D). The MUs that underwent the largest relative drop in force during this contraction were MUs 90–100 with only 40–45% of their force capacity remaining at the endurance limit.

### Submaximal force trials (80% target)

**Fig 4:**
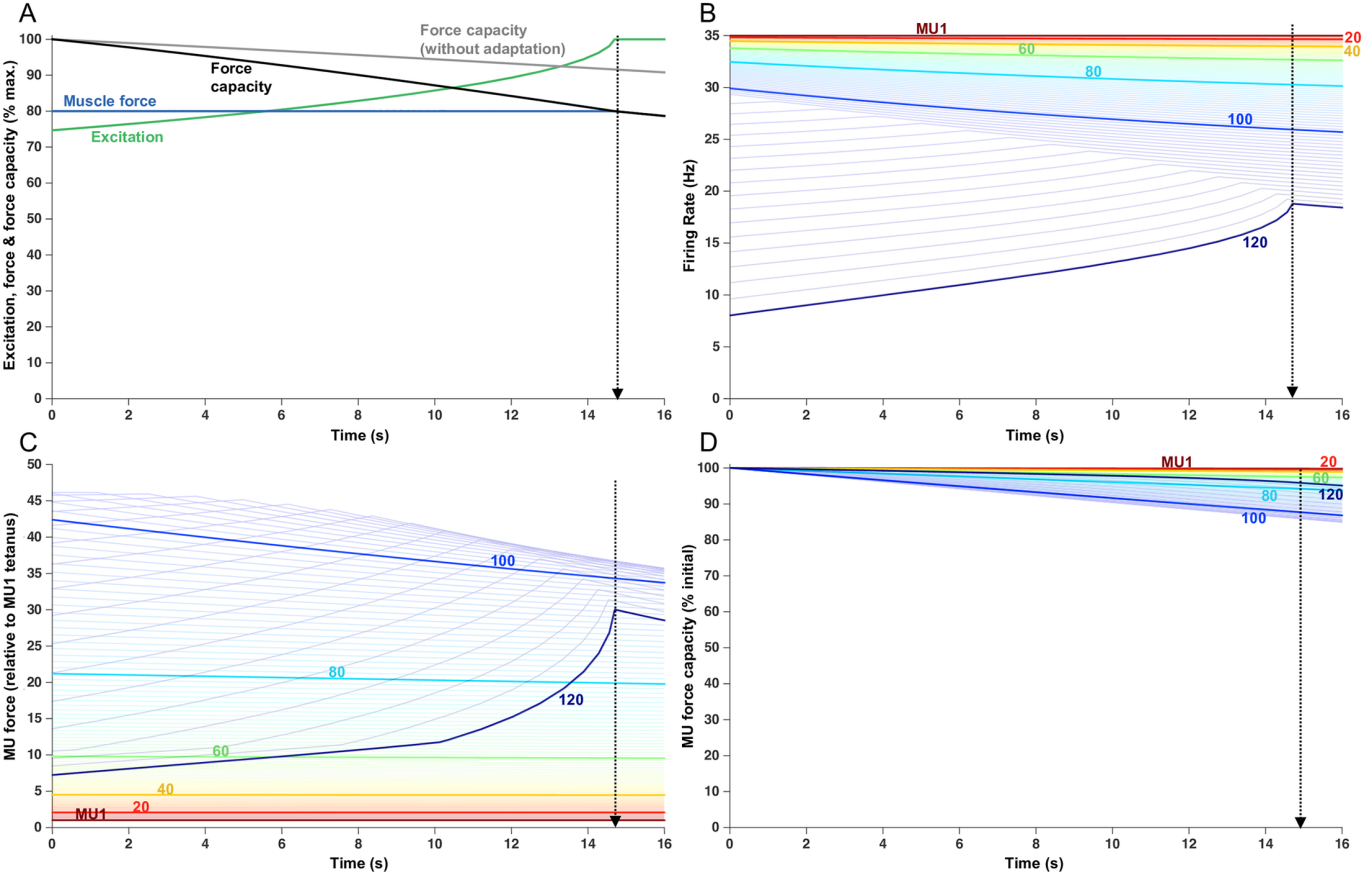
Fatigue model outputs for a sustained 80% MVC load with an endurance time of 14.8 s. (A) Increased excitation in response to fatigue. Force capacity is shown with and without firing rate adaptation and the modeled force remains at the target load until the endurance time. (B) Firing rate of each MU, over the course of the trial. Lines begin when the MU was recruited. Each 20th MU is highlighted and labelled, but all 120 MUs are shown as lighter lines. (C) Force contribution of each MU. (D) Relative force capacity of each MU (normalized to its rested capacity). Note the higher y-axis scale (50) than with the 20% MVC force (22) and 50% MVC force (30).

For comparison with the 20 and 50% contractions, Fig 4 shows simulation results for a sustained 80% force contraction. From the outset of the contraction, total muscle force capacity decreased steadily, which was counteracted by a progressive increase in excitation to maintain muscle force at the target level (Fig 4A). However, after only about 15 s of activity, maximal excitation was reached and, as such, the declining total muscle force capacity could no longer be counteracted and the endurance limit was reached. This endurance time (14.8 s) was only about 3% of that associated with the 20% force contraction and 15% of the 50% force contraction. All MUs were recruited at the start of the trial (Fig 3B). Most motor units (MUs 1–103) initiated their activities at their maximal firing rates. Consequently, their firing rates declined progressively over the trial because of the effects of firing rate adaptation, the degree of which varied as a function of MU threshold. In an experimental study, that used a similar target force as used in this simulation, MU firing rates also tended to progressively decrease with time [30]. Because the highest threshold MUs (MUs 104–120) in the simulation were initially activated below their maximal firing rates, as excitation increased, their firing rates first increased toward their maximal rates followed by a gradual decline in firing rate due to adaptation.

Due to the combined effects of firing rate adaptation and peripheral fatigue, MUs activated from the outset at their maximal rates showed a progressive decline in force (Fig 4C) with the greatest losses occurring in the strongest (and most fatigable) MUs. Motor units that were activated initially at rates below their maximum (MUs 104–120) exhibited an initial increase in force as their firing rates increased, followed by a decline in force as firing rates adapted and the process of peripheral fatigue continued. At the endurance time, the degree of force capacity loss (relative to the initial forces) was relatively small for all MUs (Fig 4D). For example, the MU exhibiting the greatest fatigue (MU107) still retained ~86% of its force capacity at the endurance time. This contrasted with the 20% force contraction (Fig 2D) in which 28% of the MUs were completely exhausted. Nevertheless, for the 20%, 50%, and 80% force contractions, the simulations indicated a complex interplay of force contributions among the MU population (Figs 2C, 3C and 4C) with individual forces increasing and decreasing, at varying times and with different rates, but with the total muscle output maintaining the target force up until the endurance limit.

### Maximum force trial (100% target)

**Fig 5:**
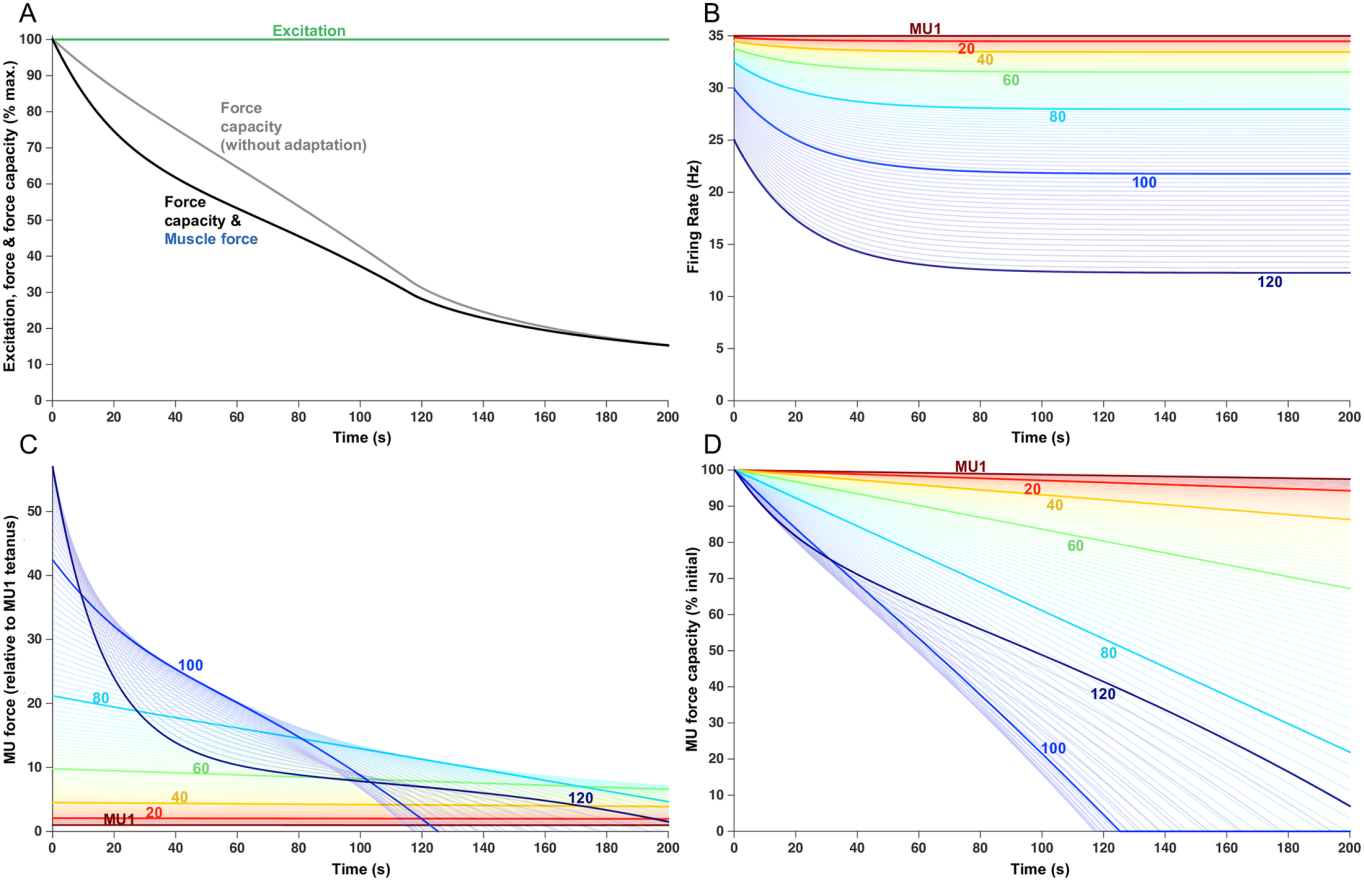
Fatigue model outputs for a sustained 100% MVC load for 200 s. (A) Increased excitation in response to fatigue. Force capacity is shown with and without firing rate adaptation and the modeled force remains at the target load until the endurance time. (B) Firing rate of each MU, over the course of the trial. Lines begin when the MU was recruited. Each 20th MU is highlighted and labelled, but all 120 MUs are shown as lighter lines. (C) Force contribution of each MU. (D) Relative force capacity of each MU (normalized to its rested capacity). Note the higher y-axis scale (57) than with the 20% MVC (22), 50% MVC (30), and 80% MVC (50) force.

Fig 5 shows simulations associated with a sustained maximum voluntary effort (100% MVE). The model predicted an immediate decrease in total muscle force capacity with an endurance time less than 1 s (Fig 5A). To mimic what has been done experimentally for such contractions, we continued the simulation out to a time of 200 s. Total muscle force declined relatively steeply over the first ~ 40 s of the contraction, then somewhat less steeply up to about 120 s, and finally with a more gradual decline in force over the last ~ 80 s of the contraction. Because voluntary excitation was maximum throughout, muscle force and force capacity were the same (Fig 5A). At the end of the 200 s contraction, force was down to ~15% of the initial force.

Because excitation was maintained at 100% throughout the contraction, changes in firing rate (Fig 5B) were caused entirely by firing rate adaptation. Fig 5B also nicely illustrates the differential effects of adaptation across the MU population, with low threshold MUs showing little adaptation and high threshold units exhibiting marked adaptation.

As expected, the inclusion of adaptation led to a greater decrease in muscle force (black trace, Fig 5A) as compared to simulations without adaptation (gray trace, Fig 5A), especially in the first 160 s, after which time there was little difference. This was a consequence of a complex interaction between normalized firing rate, normalized force (Fig 1C), instantaneous fatigability (Eq 10, Methods), and fatigue-related changes in contraction time (Eq 11, Methods). Since all MUs were recruited at the start of the trial, the effects of firing rate adaptation dominated in the first 35 s. However, because MU contraction times increased with MU fatigue, this tended to shift MUs higher on their force-frequency curves, thereby partially offsetting the force loss associated with declining firing rates due to adaptation. Consequently, the difference in the degree of force decline between simulations, that included and excluded adaptation, tended to dissipate in the latter third of the trial.

Fig 5C shows the force contribution of the individual MUs over the course of the 100% force trial. It is important to note that, at the outset of the trial, before any fatigue had occurred, the forces produced by the highest threshold MUs were less than their theoretical maximum forces. For example, MU120 had a capacity to generate 100 times more force than MU1, yet its initial force at 100% MVE was only 57 times greater than MU1. This was due to: (a) the imposed ‘onion skin’ organization that limits the maximum firing rates of high threshold MUs to be less than that of low threshold MUs, and (b) the briefer contraction times of the high threshold MUs which decreased their normalized firing rates and led to lower forces. This implies that there is a reserve capacity of force (mostly vested in the highest threshold motor units) that normally is not accessed even during maximal voluntary efforts. There is substantial circumstantial evidence that lends support to this idea [31–34].

The initial steep drop in muscle force (Fig 5A) was primarily due to the rapid loss of force occurring in the highest threshold, strongest MUs (Fig 5C). Those MUs lost force quickly because of a combination of greater firing rate adaptation and greater fatigability. After about 60 s, when firing rate adaptation was largely complete for all MUs, MU forces declined relatively steadily although with different slopes for different MUs related to their individual force capacities. An interesting exception existed with the highest threshold MUs. For example, after 60 s, the slope of force capacity decrease was less for MU120 than for MU100 (Fig 5C). This was primarily due to the initial greater extent of adaptation for MU120 than MU100, causing a larger decrease in its firing rate, which combined with its short contraction time to substantially shift it to the left on its force-frequency curve. This, in turn, led to a marked and early reduction in force output of MU120 such that it produced substantially less force than MU100 at 60 s. Because MU fatigability was partially dependent on normalized force (Eq 10, Methods), the rate of force decline was less for MU120 than MU100 for much of the contraction. The rate of total muscle fatigue decreased after ~120 s because many high threshold MUs became exhausted and could no longer further lose force capacity.

Indeed, after 200 s of sustained maximum voluntary excitation, many high threshold motor units (MU86-119) had lost virtually all their force generating capacity (Fig 5D) and MU120 generated only ~10% of its initial force. Because these high threshold MUs were also initially substantially stronger than the lower threshold units, such large losses in their force were associated with the large overall drop in total muscle force capacity during this trial.

### Endurance time predictions

**Fig 6:**
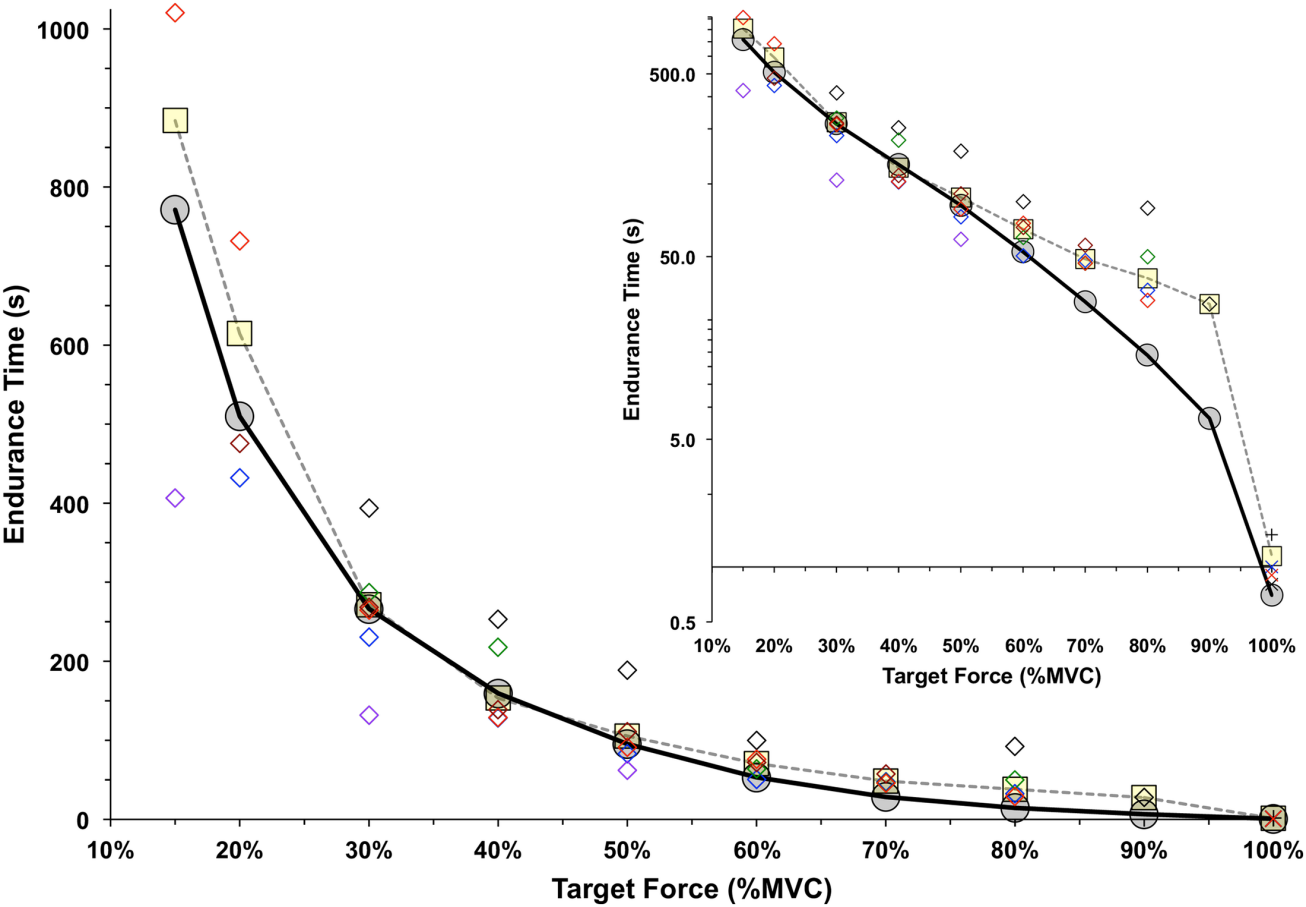
The model-predicted endurance times (grey circles) are compared to those from empirical studies (yellow squares). The endurance times summarized by Frey Law & Avin (2010) were used for contraction levels from 15% to 90% MVC, and a weighted average was calculated at each load based on the number of means involved. Open diamonds indicate the weighted averages for the ankle (black), knee (blue), trunk (green), shoulder (purple), elbow (red) and hand (brown). The data of Fig 5 were used to calculate the average duration until a 1% MVC drop with a 100% MVC load for Jones et al [39] for ankle dorsiflexors (X), Kent-Braun et al [38] for ankle dorsiflexors (+), Bigland-Ritchie et al [36] for knee extensors (blue X) and Bigland-Ritchie [37] for elbow flexors (red X). The inset graph shows the endurance times on a log scale.

Endurance times were determined for a set of simulations (like those shown in Figs 2, 3 and 4) for target force levels at 15% of maximum, and from 20–100% maximum in 10% increments. The resulting relation between predicted endurance time and target force is shown in Fig 6 (solid black line). The weighted average values of endurance times, determined experimentally for six different joints during submaximal contractions [35], and from three joints during maximum contractions [36–39] are shown in Fig 6.

Overall, there was a good correspondence between predicted and actual endurance times across a wide range of forces, joints, and studies. Across the nine efforts from 15% to 90% MVC force, the average and RMS differences between the model-predicted and empirical endurance times were -33.7 s and 52.3 s, respectively. These amounted to -3.9% and 6.0% of the full range of empirical endurance times (884 s at 15% MVC). The largest absolute difference in endurance times, between simulated and empirical values, was -112.8 s for the 15% MVC effort, representing12.8% of the empirical mean of 884 s. For the higher forces of 70, 80 and 90% MVC, there were larger relative differences between the model and empirical means (see inset, Fig 6). However, the absolute magnitude of these differences never exceeded 24 s.

### Comparison of simulated and empirical sustained maximal contractions

**Fig 7:**
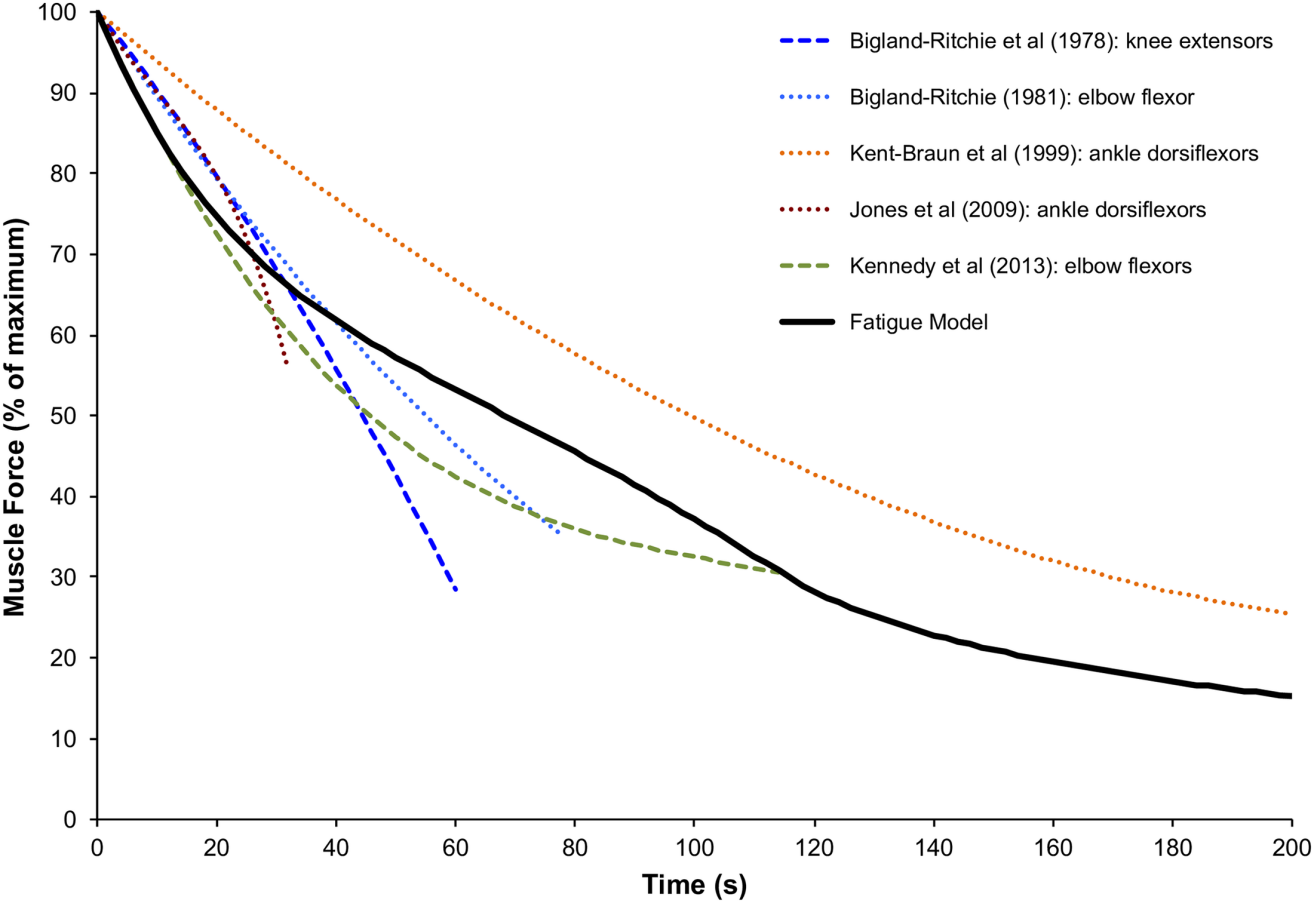
The decline in force capacity with a 100% MVC load, from Bigland-Ritchie et al [36], Bigland-Ritchie [37], Kent-Braun et al [38], Jones et al [39], and Kennedy et al. [40], are compared to the fatigue model output with and without excitation adaptation.

Fig 7 shows the simulated force and experimentally measured forces during sustained maximal contractions. In general, there was a reasonably good match between the simulated and experimental results. In the first 20 s, however, simulated force dropped somewhat more steeply (1.4% MVC/s) than that recorded experimentally (average of 1.0% MVC across the four experiments). The time at which force had dropped to 50% of maximum was ~70 s for the simulation. That time was quite similar to the ~61 s value averaged from the four experimental studies that had contractions of sufficient duration to cause at least a 50% decline [36–38,40]. The simulated loss of force beyond 20 s, and until 200 s, paralleled quite closely that of the one experimental study [38] that monitored sustained maximal contractions for 200 s. Nevertheless, the force at 200 s in that study was about 25% of the initial force, whereas the simulated force at that time was ~16% of the initial force.

### Multiple force plateaus

**Fig 8:**
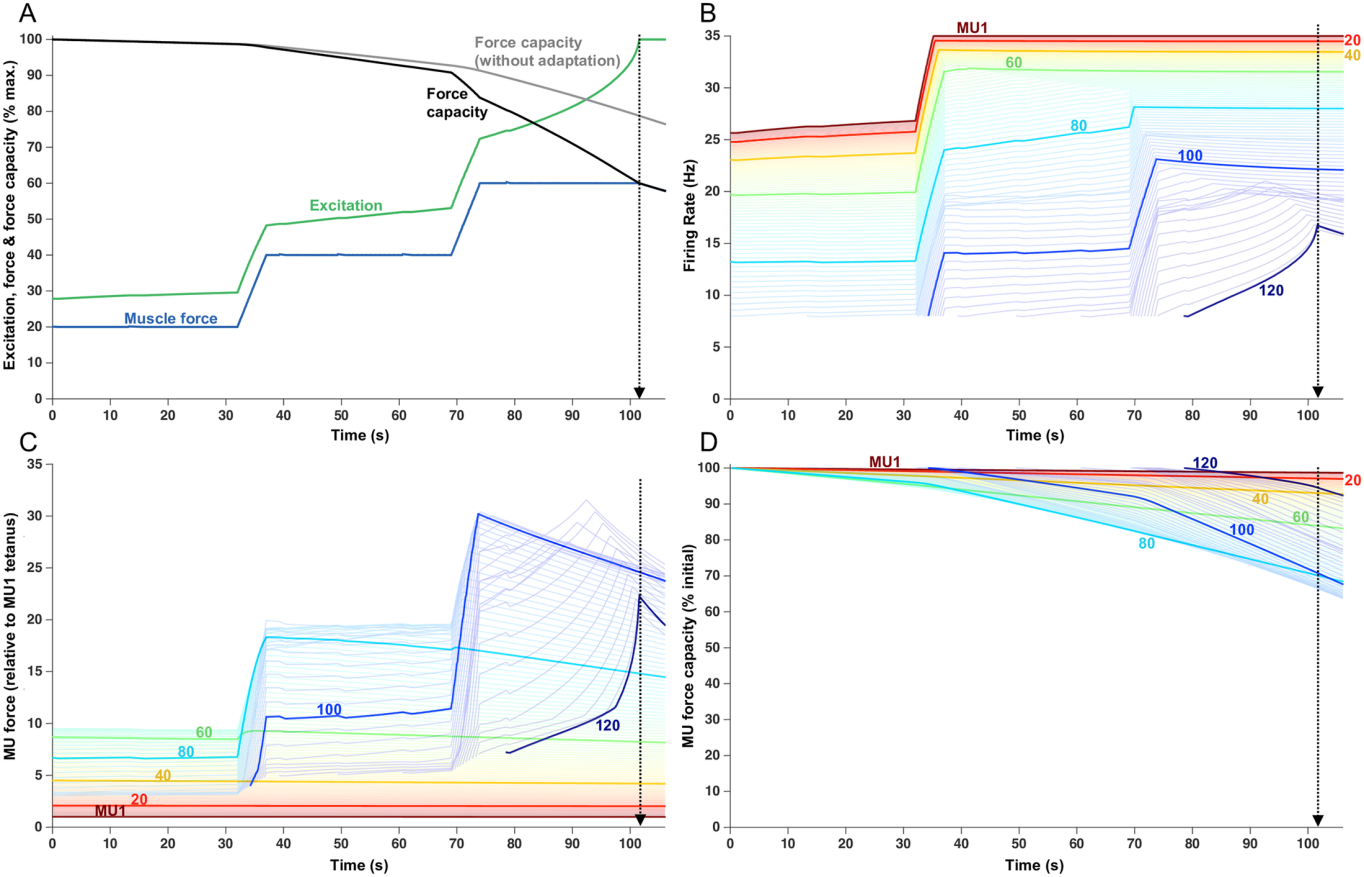
Fatigue model outputs for a series of progressively higher force plateaus, involving 32 seconds of 20, 40 and 60% separated by 5 s linear ramps from one level to the next. Endurance time was 101.5 s. (A) Increased excitation in response to fatigue. Force capacity is shown with and without firing rate adaptation and the modeled force remains at the target load until the endurance time. (B) Firing rate of each MU, over the course of the trial. Lines begin when the MU was recruited. Each 20th MU is highlighted and labelled, but all 120 MUs are shown as lighter lines. (C) Force contribution of each MU. (D) Relative force capacity of each MU (normalized to its rested capacity).

Given the reasonable correspondence between simulated force and experimental findings, we were encouraged to carry out further simulations involving somewhat unconventional tasks to highlight the potential of the model to predict fatigue under more complex circumstances. Fig 8 shows a simulation involving a ‘staircase’ task in which the force was maintained for 32 s at progressively increasing 20% MVC plateaus with a brief ramp increase in force between plateaus. The endurance time for this task was 101.5 s and occurred during the third plateau when the 60% MVC target could no longer be maintained (Fig 8A). The first plateau (20% MVC) was maintained with very little change in force capacities of the active MUs (Fig 8C) requiring only a subtle increase in excitation. As such, the firing rates of the MUs active during the first plateau (MUs 1–90) changed little (Fig 8B) and only one new unit (MU91) was recruited (at ~15 s into the trial).

The increase in excitation necessary to attain the second plateau (40% MVC) was accompanied by ~13 imp/s increase in firing rate in MUs that were active during the first plateau (Fig 8B), plus recruitment of an additional 15 MUs (MUs 92–106). However, this increase in firing rate had little effect on force generated by the lowest threshold MUs (Fig 8C) because their firing rates were already high enough to place them on the plateau of the force-frequency curve (Fig 1C). For example, during the transition from the 20% to 40% plateau, MU40 started with a contraction time of ~63 ms and increased its firing rate from 23.7 to 33.6 imp/s. At 23.7 imp/s, the normalized firing rate is 23.7 imp/s x 0.063 s = 1.49, which is associated with a force output of ~100% of maximum for that MU (Fig 1C). As such, increasing the firing rate to 33.6 imp/s had a negligible effect on force for MU40. However, the increased firing rate of higher threshold (and faster contracting) MUs (e.g. MU80), did translate into marked increases in force. Those increases, combined with the recruitment of higher threshold (and stronger) MUs, enabled the 40% target to be attained. Those units contributing the greatest amount of force during the 40% plateau were also relatively more fatigable (e.g. MU 80). As their force started to decline during the sustained 40% plateau (Fig 8C), excitation progressively increased (Fig 8A). The increased excitation caused firing rates to increase in those MUs (60–106) that had not yet reached their maximal firing rates (Fig 8B). It is interesting to note that the slope of the firing rate increase varied systematically across these MUs during this time (Fig 8B). The lower threshold MUs (e.g. MU 80) had steeper slopes than higher threshold MUs (e.g. MU100). This was a consequence of firing rate adaptation being greater for higher threshold MUs, compared to lower threshold MUs, which more potently attenuated the increases in firing rate in these MUs during increased excitation. The increased excitation during the 40% plateau also led to the recruitment of three additional MUs (Fig 8B).

The increase in excitation needed to achieve the 60% MVC target resulted in increases in firing rates in those MUs that had not yet saturated (MUs 72–109) and the recruitment of all the remaining MUs but MU120 (Fig 8B). Force then dropped off relatively steeply in some high threshold MUs (e.g. MU100, Fig 8C) because these MUs were assigned high fatigability values (Eq 9) and they were no longer capable of increasing firing rate. This force loss was partially compensated for by increasing firing rates of the most recently recruited MUs and the recruitment of the last unit (MU120, Fig 8B). The increases in firing rate in these high threshold, strong and highly fatigable MUs led to an initial and brief escalation in their force (Fig 8C) followed by a steep decline, such that the muscle's maximum force capacity eventually fell below the target force (at ~102 s). The MUs most impaired by this task, in terms of loss in force capacity at the end of the trial, were MUs 66–101 (Fig 8D). There were no exhausted MUs and the MU exhibiting the greatest fatigue (MU93) still retained ~65% of its force capacity at the endurance limit.

### Three initial forces to the same fatigue level

A second set of somewhat unconventional tasks involved using target forces of 15%, 50% and 85% MVC but, in each case, the simulation was continued until muscle force had decreased below15% of maximum. As such, each case was associated with the same degree of total muscle fatigue (ie. 85%, as conventionally defined) but brought about by different ‘paths’. We were interested to know whether these different routes to the same level of total muscle fatigue would have differential effects on the MU population.

**Fig 9:**
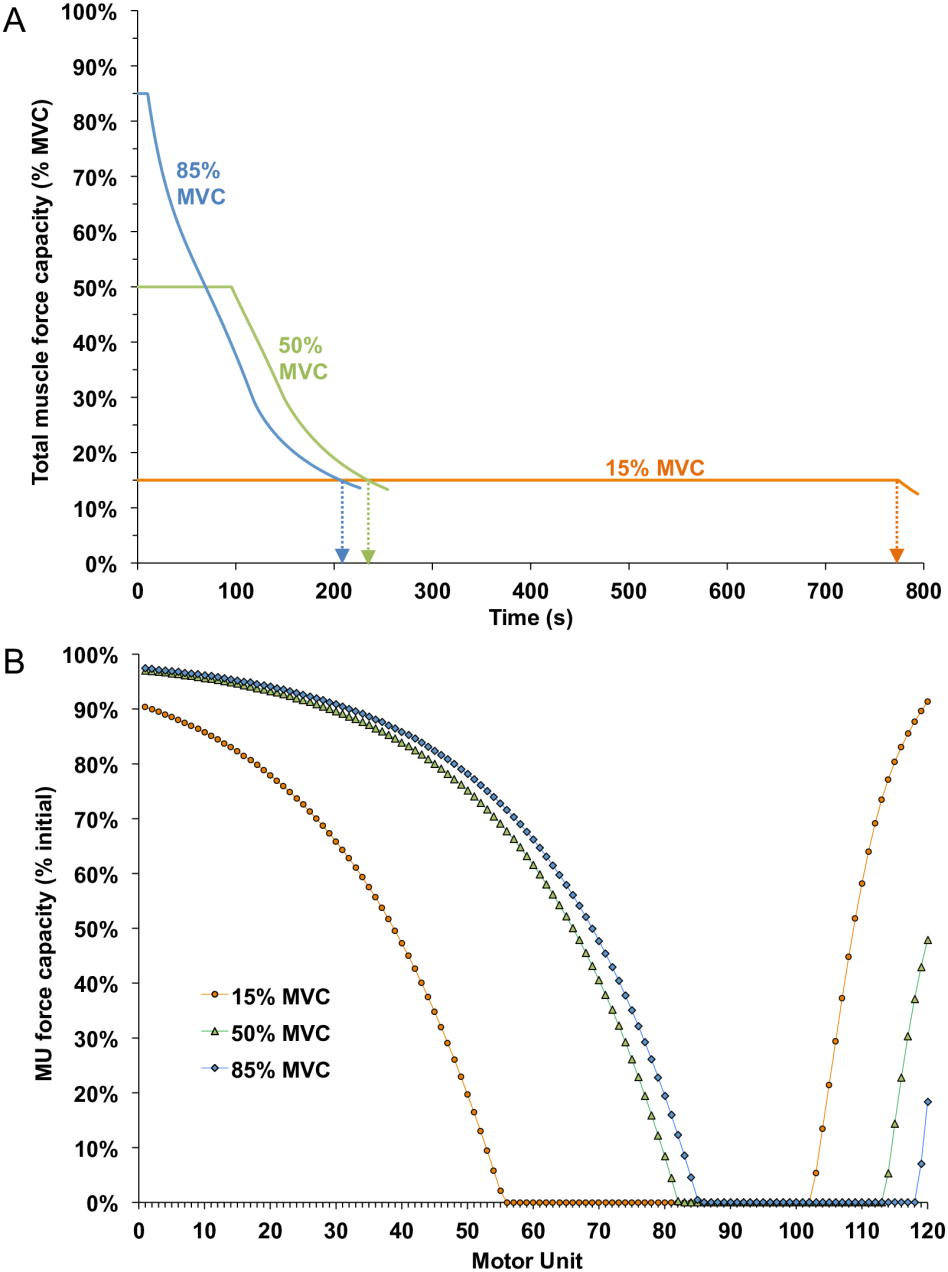
The total muscle and motor unit capacities for initial target forces of 15, 50 and 85% until total muscle capacity decreased to 15% MVC (ie. 85% muscle fatigue for each trial). (A) Fatigue model outputs for total muscle capacity. Arrows indicate when force fell below 15% MVC. (B) Final force capacity of each MU, normalized to its rested capacity, when total muscle capacity reached 15% MVC for each initial force condition (shown with a vertical arrow in 9A).

As shown in Fig 9A, force was maintained at the 85% MVC target (blue trace) for only about 10 s before declining and eventually dropping to 15% MVC at a time of 206.5 s. Likewise, force was maintained at the 50% target (green trace) for about 95 s before decaying to 15% MVC at a time of 234.5 s. For the 15% MVC target (red trace), force was maintained at that level for 774.0 s. Fig 9B shows the remaining relative force capacity of each MU, at the time force dropped below the 15% MVC target, for each of the three cases. Despite the same 85% decrease in muscle force capacity, the profiles of fatigue across the MU population were strikingly different for the three cases. The 15% target trial (red symbols) resulted in substantially greater fatigue in the lower threshold MUs compared to the other two cases, but with less fatigue in the highest threshold MUs. On the other hand, for the 85% target trial (blue symbols), the degree of fatigue was greater (i.e. lower force capacities) for the high threshold MUs as compared to the other two cases, but with less fatigue in the lower threshold MUs. In addition, the sets of MUs that became completely exhausted differed for the three different cases: MUs 56–102, MUs 83–113, and MUs 86–118 for the 15%, 50%, and 85% target force trials, respectively.

## Discussion

We developed a model to predict the time course of total muscle fatigue based on the changing force capacities of individual MUs in response to a wide range of task demands. The method was based on a MU population model [19] to which peripheral and central fatigue effects were added. The model accurately estimated endurance times for sustained isometric contractions across a wide range of target levels (Fig 6). In addition, simulations were run for situations that have little experimental precedent to demonstrate the potential utility of the model to predict motor unit fatigue for more complicated, real-world applications. Moreover, the model provided insight, into the complex orchestration of MU contributions during fatigue, that would be unattainable with current experimental approaches and, perhaps, difficult to envision based on detailed knowledge of the physiological properties of individual MUs.

### Comparison with other models

Many models have been published that predict the mechanical aspects of muscle fatigue [41–43]. For example, the three-compartment model of muscle fatigue developed by Liu et al. [43] has been used effectively by several investigators to predict fatigue for a variety of tasks (e. g. [44–47]). However, that approach essentially simplifies muscle physiology to one type of MU and assumes MUs are fully rested, fully activated, or completely fatigued.

Other models have been developed that predict the responses of groups of MUs (e.g. [48,49]) or individual MUs [50–52]). For example, the Dideriksen model [50] was a pioneering effort that used changes in metabolite concentrations within muscle as a key factor driving alterations in MU contractility and neural drive during fatigue. Such a mechanistic model was necessarily complex and, as such, the output of the model generally presented only the net effect of prolonged activity on total muscle capacity. Because our model was less complex, while still accounting for individualized responses for an entire MU population, it could make accurate predictions about total muscle fatigue (Fig 6) and readily display the interplay of force contributions among the constituent MUs during a wide variety of fatiguing tasks (Figs 2, 3, 4, 5 and 8).

There were also some differences in the physiological representations of the Dideriksen et al. [50] model as compared to the present model. The Dideriksen model employed a ‘cross-over’ scheme to predict MU firing rates, wherein higher threshold MUs ultimately discharge at higher rates than low threshold MUs. While there are some data to support this type of organization [53,54], many findings suggest a nested ‘onion-skin’ organization (as used here) in MU firing rate profiles [16–18,23,55,56]. Another difference between the two models is in the degree of fatigability of different MUs. In the Dideriksen model, none of the MUs would have been classified as ‘fatigable’ according to conventional criteria of Burke et al. [10] (i.e. fatigue index values < 0.25). In our model, the highest threshold and most fatigable MUs had fatigue-index values as low as 0.1, consistent with the original data of Burke et al. [10]. However, questions remain as to how well data obtained from cat hindlimb MUs (e.g. [10]) might generally represent the properties of human MUs [9,57,58].

A third distinction between the two models relates to the implementation of adaptation of firing rates during sustained activity. The Dideriksen model did not account for intrinsic changes in excitability of motor neurons associated with spike frequency adaptation. Such adaptation, however, is a well-established property of motor neurons [59–69]. Furthermore, such intrinsic changes in excitability may partially explain the observed differences in firing responses across MUs (i.e. some with decreasing firing rates while others are recruited and increasing their firing rates) during fatiguing contractions that would be otherwise difficult to account for with broadly distributed sources of synaptic input [25,29,70].

### Predictions

The ability of the model to reveal the intricate interplay of MU contributions during fatigue provided interesting predictions and insights. For example, the model predicted that low-force contractions, sustained to their endurance limit, induce more fatigue across the MU population than high-force contractions (compare Figs 2D and 4D). This prediction has implications for rehabilitation medicine, as it suggests that relatively weak contractions could provide a potent exercise stimulus for much of the MU population without the risks associated with intense, high-force contractions.

In addition, the model predicted that loss in force capacity was always more pronounced among the upper-middle range of motor units (from ~ MU60 –MU 110) while both the lowest threshold and highest threshold MUs subpopulations were less impaired across a wide range of tasks (Figs 2D, 3D, 4D, 5D, 8D and 9B). Relative to low threshold MUs, the greater fatigue in the upper-middle range of MUs occurred simply because they were intrinsically more fatigable than the low threshold MUs, while being active for practically the same durations. On the other hand, greater fatigue in the upper-middle range of MUs, compared to the highest threshold MUs, was due to their more prolonged involvement in many of the tasks (e.g. Figs 2B, 3B and 8B) and because they tended to sustain higher levels of absolute force (provoking greater fatigue) than the highest threshold MUs (e.g. Figs 4C and 5C). The highest threshold MUs, despite their intrinsic capability to generate the largest forces, never achieved their full force capacities because of the limits placed on their maximum firing rates. The physiological mechanisms underlying such firing rate saturation are not yet known despite several recent investigations into this phenomenon [71–75]. In addition, firing rates decreased more precipitously for the highest threshold MUs than other MUs (e.g. Fig 4B) due to greater firing rate adaptations. Such reductions in firing rate led to lower forces and, thereby, lessened their fatigue compared to the upper-middle threshold group of MUs.

Another prediction involved simulations of sustained 15%, 50% and 85% MVC force. The model predicted marked differences in MU fatigue when the total force capacity decreased to the *same* level of 15% MVC (i.e. 85% fatigue) (Fig 8). Although all three cases led to large subsets of MUs that were exhausted, the specific MUs in each subset differed depending on the initial target force. Furthermore, the degree of fatigue, in those units still capable of producing force at the endurance limit, varied substantially across the three conditions. Therefore, despite an equivalence in the degree of muscle fatigue, based on the prevailing definition of fatigue (a reduction in muscle force/power capacity), the physiological status of the motor unit population was quite different under the three conditions. Such differences could have important implications, for example, in determining the subpopulations of MUs receiving the greatest exercise stimulus in the context of strength or endurance training and for how the muscle responds to ensuing demands and recovery in the context of physical work occurring within an industrial setting.

The recent development of high-density multi-electrode arrays [76–79], combined with sophisticated decomposition algorithms [55,80,81], enables tracking of a large numbers of MUs during a wide range of contractions (e.g. [56,82,83]). Such technology should make it possible to evaluate some of the predictions made here, particularly with regards to patterns of MU activity. Unfortunately, however, few methods are presently available that can readily measure changes in force capacity of MUs during fatiguing contractions.

### Limitations

One limitation of the present model is that it did not account for differences in the constellation of MU properties making up different muscles and/or occurring in different individuals. Instead, we opted here for a ‘one size fits all’ approach that, nevertheless, did make good predictions of endurance times for a wide range of muscles (Fig 5). However, because the model is flexible and all parameters are readily altered, one could easily carry out simulations of fatigue associated with different types of situations, such as might occur with muscles having different fiber type compositions, particular neuromuscular diseases, or with aging.

Another limitation of the present model is that it simulates isometric force only. This is a critical limitation, as most behaviors involve dynamic muscle activity. This is an especially challenging limitation to overcome because there are so little experimental data involving lengthening and shortening contractions in individual MUs. In this regard, perhaps mechanistic models of fatigue (e.g. [50,52]) combined with Hill-type models of contractile dynamics MUs [84] could, from first principles, make good predictions about fatigue arising during tasks involving movement.

A further limitation of the present model is that we used only one of a number of neural mechanisms that can contribute to central fatigue (see [6]). For simplicity, diminished intrinsic excitability (associated with firing-rate adaptation) served as a representative mechanism underlying central fatigue. As such, fatigue-related alterations in descending drive (e.g. impaired motor cortical output) and sensory feedback (e.g. increased inhibition associated with activation of metabolite-sensitive receptors in muscle) were not explicitly simulated in the model. Nevertheless, there is a significant body of experimental work that has concluded, for example, that feedback from metabolite-sensitive receptors does not appear to significantly inhibit motor neurons during fatigue [85–88]. In addition, reduced excitability of motor neurons during fatigue does not appear to be due to diminished peripheral excitatory input [89]. Furthermore, some evidence indicates that the motor cortex is relatively unimpaired during voluntary fatiguing contractions [90]. On the other hand, there is compelling data indicating diminished *intrinsic* excitability of motor neurons during fatigue [91]. As such, it seemed reasonable to use reduced intrinsic excitability as a proxy for central fatigue in the present model. However, there are sure to be differences in the relative contributions of various central fatigue mechanisms that depend on the muscle group involved (e.g. [92]) or task [70,93].

And finally, an additional limitation with the present version of the model is that does not include recovery from fatigue. In real world situations, muscle fatigue usually does not occur in isolation—it is influenced by previous bouts of muscle activity and the degree of intervening rest. This issue is particularly relevant to physical ergonomics, a field that has produced many analysis tools to determine the acceptability of an isolated task, but almost no methods are available to estimate muscle fatigue and injury risk associated with the typical case of workers performing a combination of different subtasks, including brief periods of rest, as part of their whole job. In its next version, our fatigue model will be expanded to include both the fatigue *and* recovery of MUs, so that the effects of combined efforts can be assessed. This addition could assist in determining the acceptability of whole jobs and/or for optimizing task allocation and sequencing. The model could also then be used to design exercise and rehabilitation programs that set demand magnitudes and work/rest ratios to optimize the exercise stimulus, given the particular physiological state of the motor unit population.

## Material and methods

An existing MU population model was used to simulate MU firing rates and isometric muscle forces (see [19] for details). To that model, we added fatigue-related changes in MU force, contraction time, and firing rate associated with sustained voluntary contractions. Our goal was to develop a tractable model that could be readily implemented to estimate changes in overall force capacity of the whole muscle and of 120 individual MUs associated with a wide range of fatiguing tasks, including those relevant to exercise protocols, occupational tasks, and rehabilitation programs. As such, rather than model the specific (and numerous) cellular processes governing fatigue-related adaptations (e.g. [50,52,94,95]), we simulated changes in MU and muscle properties based largely on those that have been described empirically. The model was implemented in the MATLAB environment (The MathWorks, Natick, MA) and the code can be downloaded at: https://goo.gl/Frmw8w. The authors can be contacted for further information and/or updates to the model or code.

### Motor unit pool

The model presented here represents only one of many plausible schemes to simulate MU activity and force. Consequently, parameter selections were meant to be generally representative, but not definitive, characterizations of any specific skeletal muscle. The model enables users to readily specify parameters needed to simulate a variety of MU organizations. For the present study, the simulated muscle consisted of a pool of 120 MUs. MU twitches were modeled as the impulse response of a critically damped 2nd order system (Fig 1A). Each MU, *i*, was assigned a unique twitch amplitude and twitch contraction time. The distribution of MUs based on twitch amplitude, *P*, was determined using the exponential function [19]:

$$\text{P}\left(\text{i}\right)\;={\text{ e}}^{\left[\text{ ln}\left(\text{RP}\right)\;\left(\text{i }-\;1\right)\;/\;\left(\text{n}-1\right)\;\right]}$$ (1)

where *ln* is the natural logarithm, *RP* is the desired range of twitch forces across the pool, and *n* is the number of MUs in the pool (i.e., 120). For these simulations, *RP* was assigned a value of 100. Such a representation yields a distribution with many weak MUs and relatively few strong MUs. Maximum MU forces were normalized to the force of MU(1) such that the force of MU(1) was 1.0 and MU(120) was 100.0 force units. Contraction times were assigned as an inverse function of twitch amplitude (see [19]) for specific formulation) and ranged from 30 ms for the strongest unit, MU(120), to 90 ms for the weakest, MU(1) (Fig 1B).

All MUs within the pool received the same level of excitatory drive (*E*) that could vary as a function of time (*t*). The amount of excitatory drive needed to recruit each MU, referred to as 'recruitment threshold excitation' (*RTE(i)*), was also determined as an exponential function that assigned many MUs to have low thresholds and few to have high thresholds, using:

$$\text{RTE}\left(\text{i}\right)\;={\text{ e}}^{\left[\text{ ln}\left(\text{RR}\right)\;\left(\text{i }-\;1\right)\;/\;\left(\text{n}-1\right)\;\right]}$$ (2)

where *RR* is the desired range of recruitment thresholds and was set to 50 for the present simulations. A MU, therefore, was recruited when the excitatory drive equaled or exceeded its assigned recruitment threshold excitation (*RTE(i))*. Therefore, and in general accordance with the size principle, weaker MUs (i.e. those with low twitch forces) were recruited at lower levels of excitation than stronger MUs.

At threshold excitation, MUs discharged at a minimum firing rate (*minR*) of 8 impulses (imp)/s. Firing rate (R) increased linearly with increased excitation up to an assigned maximum rate (maxR(i)) for each MU, beyond which no further increases in rate occurred (i.e. firing rate saturated). The slope (i.e. the gain, *g*) of increased firing rate with excitation was set to be the same for all MUs (1 imp/s for each unit increase in excitation) and firing rate was modeled as:

R(i,t) = g[E(t)− RTE(i)]+ minR (3)

Based on considerable experimental findings, maximum firing rates (*maxR(i)*) were modeled as an inverse function of recruitment thresholds, yielding a nested or ‘onion skin’ arrangement of firing rates across the MU population (e.g., [18,56]). In the present model, *maxR* was assigned to be 35 imp/s for MU(1) and decreased uniformly to 25 imp/s for MU(120). For excitation levels above that needed to bring a MU to its assigned maximum rate, firing rate was maintained at *maxR(i)*. Maximum excitation (*E*_*max*_) to the MU pool was defined as the amount of excitation needed to bring the highest threshold MU to its assigned *maxR(i)*. Rearranging Eq 3 to solve for the excitation (E) associated with this situation yields: E_max_ = RTE(120) + (maxR(120)—minR)/g = 50 + (25–8)/1.0 = 67 excitation units, such that the last MU is recruited at 50/67 = 74.6% of *E*_*max*_.

### Force-frequency relation

The relation between MU (or whole muscle) force and firing frequency generally exhibits a sigmoid form [96,97]. The specific shape of the force-frequency relation depends on contractile speed, in that MUs with long duration contraction times attain tetanic fusion (i.e. plateau on the sigmoid) at lower rates than do MUs with brief contraction times [98–100]. If, however, the MU stimulus frequency or firing rate (*R(i*,*t))* is normalized to the inverse of the twitch contraction time (1/*CT(i)*), force-frequency curves are similar for most MUs [19,99]. The normalized firing (or stimulus) rates (*NR*) can be represented as:

NR(i,t) = R(i,t)/ [1/CT(i)] = R(i,t) x CT(i) (4)

A composite linear and sigmoidal relationship (see Fig 1C) was used to estimate normalized force (*NF)* as a function of normalized firing rate (*NR*), as originally derived by Fuglevand et al [19] but simplified to:

for NR(i,t) ≤ 0.4,  NF(i,t) = 0.3 NR(i,t) (5)

$$\text{for NR}\left(\text{i},\text{t}\right)\;>\;0.4,\text{ NF}\left(\text{i},\text{t}\right)\;=\;1-{\text{e}}^{-2\text{ NR}{\left(\text{i},\text{t}\right)}^{3}}$$ (6)

The instantaneous force (*F*) of each MU was then scaled as a function of its assigned twitch force, *P(i)*:

F(i,t) = NF(i,t) P(i) (7)

The total muscle force was then calculated simply as the linear sum of all 120 individual MU forces at any given time.

### Fatigue—Peripheral factors

#### Force capacity

There are numerous factors that can diminish the force capacity of individual MUs during prolonged activity [1–3,6,7,101,102]. Furthermore, the relative contributions of these various factors to fatigue depend on the characteristics of the task and the type of MU [70,93,103]. As such, it is difficult to represent the nuance and complexity of fatigue while also keeping the parameter space of a model within reasonable limits. To strike a balance between physiological fidelity and tractability, we lumped fatigue factors into one of two general categories: peripheral and central. Peripheral factors include those affecting function of the axon, neuromuscular junction, sarcolemma, excitation-contraction coupling, and the contractile apparatus itself. Central factors refer to those that affect the excitability of, and synaptic input to, motor neurons.

Peripheral factors are selectively challenged when motor axons or motor neurons are artificially activated with repetitive pulses of electrical stimulation. Burke and colleagues [10] carried out one of the most thorough examinations of such peripheral fatigue across a population of MUs in a mammalian muscle (cat medial gastrocnemius). In that study, isometric force was recorded from individual MUs during repetitive intermittent stimulation (1 train/s, 40 Hz train, 33% duty cycle) for several minutes. Fatigue resistance (fatigue index) was quantified as the ratio of force produced at 2 minutes of stimulation to that at the outset of the fatigue protocol. Across the population of MUs, there was a large range of fatigue resistances. In general, MUs that produced the weakest tetanic forces were markedly fatigue resistant whereas the strongest MUs exhibited substantial fatigability.

For purposes of our model, 'fatigability' will refer to the rate of MU fatigue and this was based on the data of Burke et al. [10]. To do this, Burke's fatigue-index (FI) values were converted into an average percent force loss per unit time using the following:

fatigability = [(1.0 – FI) / 2 min]×100 (8)

From Fig 5 of Burke et al. [10], we estimated that the weakest MU had a fatigue index value of ~0.995 (ie. 0.5% drop in capacity) whereas the strongest MU had a fatigue index value of ~ 0.10 (ie. 90% drop in capacity). Using Eq 8, this yielded fatigability values of 0.25%/min (or 0.0042%/s) for the weakest MU and 45%/min (or 0.75%/s) for the strongest MU. This represents a 180-fold range of fatigability across the population.

One challenge related to directly applying such fatigability values in the present model was that the Burke study involved intermittent excitation whereas, here, we were simulating fatigue associated with sustained contractions. Generally, fatigue (when calculated over the entire task duration) increases with duty cycle for intermittent contractions and is greatest for sustained contractions for both single motor units [104,105] and for whole muscle [106–109]. To adjust fatigability values derived from intermittent contractions (i.e. Burke et al. [10]) to that associated with continuous contractions, we used a statistical model [110] that predicts fatigability for any combination of contraction intensity and duty cycle. Looft’s model was based on a meta-analysis of 47 human fatigue studies involving intermittent and sustained isometric contractions of ankle, knee, elbow, and hand muscles. Using duty cycles of 100% (i.e. sustained contraction) and 33% (i.e. Burke fatigue protocol) and a contraction intensity of 100% (i.e. maximal tetanic contraction), the Looft model predicted that fatigue would be 3.1 times greater for a sustained contraction compared to one that has a 33% duty cycle. Consequently, we simply multiplied the fatigabilities of the weakest and strongest MUs in Burke’s data by 3 to obtain fatigability values of 0.0125%/s and 2.25%/s, respectively, to represent the *nominal* fatigabilities associated with continuous maximal contractions.

Nominal fatigabilities (*FAT*) were assigned to each MU(i) in the modeled population such that weak (early recruited) MUs had low fatigability and strong (later recruited) MUs large fatigability using:

$$\text{FAT}(\text{i})=\text{FAT}(1)\;\times \;{\text{e}}^{\left[\text{ln}(\text{RFR})\;(\text{i}-1)\;/\;(\text{n}-1)\right]}$$ (9)

where FAT(1) is the nominal fatigability of the first MU (0.0125%/s) and *RFR* is the range of fatigabilities across the entire population (180 fold). Fig 1D shows nominal fatigabilities (from Eq 9) plotted as a function of twitch forces (from Eq 1) for all MUs in the modeled population.

For an individual MU, fatigability will likely depend also on its instantaneous force relative to its own maximum force capacity. In whole muscle, higher frequencies of stimulation (that produce higher forces) are known to provoke greater rates of fatigue than lower stimulus frequencies [111,112]. Importantly, fatigue rates, evoked using different stimulus frequencies (50 Hz and 80 Hz) that initially produce the *same* level of force (both producing near maximal force, i.e. a normalized force of ~1.0), are practically the same[112]. Such findings imply that fatigue rate is at least roughly related to normalized force. At the level of single MUs, Sandercock et al. [105] continuously stimulated cat hindlimb MUs at one of two frequencies: 10 Hz and 80 Hz. Based on pre-fatigue force-frequency curves (provided only for fast twitch MUs), 10-Hz stimulation initially produced a normalized force of about 0.05 whereas 80-Hz stimulation produced a normalize force of about 0.9, an ~18 fold difference [105]. The fatigue rate for a fast MU during continuous10-Hz stimulation (estimated from Fig 2C, [105]), was ~ 0.47% /s whereas during 80-Hz, the fatigue rate was estimated as 8%/s, ~17 times higher than at 10 Hz. Therefore, at least for this limited sample, fatigue rate was closely related to the normalized force exerted at the outset of the contraction.

Consequently, the *instantaneous* fatigability was calculated in the model as the product of the assigned nominal fatigability (Eq 9) and the normalized force (*NF*, Eqs 5 and 6, Fig 1C) developed by a MU, which, in turn, depends on the instantaneous firing rate and contraction time of the unit (Eq 4), as:

FAT(i,t) = FAT(i) x NF(i, t) (10)

#### Contraction time

Sustained contractions not only can lead to a loss in force capacity but also can contribute to a decrease in contractile speed as reflected in an increase in twitch contraction time (CT). Such changes in CT alter the normalized firing rate (Eq 4), which in turn changes the level of force exerted by a MU (Fig 1C). Because fatigability depends on the instantaneous force exerted by a MU (Eq 10), it was important to model fatigue-related changes in contraction time.

Unfortunately, there are little data characterizing changes in MU CT associated with sustained (or intermittent) fatiguing contractions. The information that is available generally indicates that MUs that exhibit the greatest force loss also show the greatest slowing in contractile speed [11,113,114]. To simulate changes in CT during fatigue for the present model, we relied on data from Shields et al [115] that showed changes in twitch CT and tetanic torque during 180 s of electrical stimulation in acutely and chronically paralyzed human soleus. Their previous study [116] showed that acutely paralyzed soleus retains properties similar to type S (early recruited) MUs, whereas chronically paralyzed soleus takes on contractile properties more analogous to type FF (later recruited) MUs. We developed regression equations based on the data of Shields et al. (their Table 1, [115]) to predict increases in CT as a function of fatigue-related decreases in tetanic torque for both groups. While there were substantial differences in the extent of torque loss and changes in CT during the 180-s bout of stimulation for the two groups, the regression equations were virtually identical. As such, we combined the data from the two groups to obtain the simple relation:

% CT = 0.379 × % FL (11)

where % CT is the percentage increase in contraction time associated with a given percentage force loss (% FL) for any MU. For example, if a MU with an initial CT of 50 ms loses 20% of its force, then Eq 11 would predict a 0.379 x 20% = 7.6% increase in CT to a value of 53.8 ms.

### Fatigue—Central factors

Central fatigue encompasses a host of mechanisms that can curtail the spiking output of motor neurons [4–7]. One category of such mechanisms is that related to diminution of net excitatory drive to motor neurons. This can occur due to decreases in excitatory input (e.g., from supraspinal centers) and/or increase in inhibitory inputs (e.g. via peripheral receptors and their spinal interneurons). In the model, such a reduction in net excitation could be implemented by decreasing the excitatory drive function, E(t), or by reducing the maximum excitation, E_max_ (Eq 4). For simplicity in the present simulations, however, neither E(t) or E_max_ were explicitly reduced to simulate this category of central fatigue mechanisms.

Another category of mechanisms underlying central fatigue are those intrinsic to motor neurons that contribute to time-dependent decreases in motor neuron firing in the presence of constant excitatory drive, referred to as firing-rate (or spike-frequency) adaptation. Here we simulated firing-rate adaptation using an approach similar to that described previously in detail by Revill and Fuglevand [117]. In brief, an exponentially rising outward (i.e. inhibitory) “current” was subtracted from the excitatory drive function to yield the net excitation acting at the spike initiation zone of a motor neuron. The extent of this intrinsic adaptation current, *A*, for any MU(*i*), was a function of both the time since MU recruitment, *TR(i)*, and the excitation level, *E(t)*, namely,

$$\text{A}\left(\text{t},\text{E}\right)=\text{ q}\left(\text{i}\right)\;\left[1-{\text{e}}^{-\left(\text{t}-\text{TR}\left(\text{i}\right)\right)/\tau }\right]$$ (12)

where τ is the time constant. We assigned the time constant a value of 22 s based on experimental observations of Sawczuk et al. [63] and Gorman et al. [60].

The parameter *q(i)* in Eq 12 designates the maximum value of the adaptation (inhibitory) current for each MU. Because the magnitude of adaptation tends to be larger with greater levels of depolarizing current and firing rate [59,60,63], *q* was modeled to depend on a MU's firing rate in excess of its minimum firing rate [i.e., *R(i*,*t)—minR*]. In addition, the magnitude of firing-rate adaptation appears to be more pronounced in high threshold compared to low threshold MUs [59,64]. Therefore, we also included recruitment threshold as an additional factor influencing the maximum extent of adaptation, *q* for each MU, using:

$$\text{q}\left(\text{i}\right)=\phi \;\left[\text{R}\left(\text{i},\text{t}\right)\;-\text{ minR}+\text{ d}\right]\;\left[\frac{\text{RTE}\left(\text{i}\right)\;-\;1}{\text{RTE}\left(\text{n}\right)\;-\;1}\right]$$ (13)

where (RTE(i)—1)/(RTE(n)—1) indicates the recruitment threshold excitation of any MU(i), relative to the largest threshold MU(n), or RTE(120) in our model. The parameter ϕ was selected to match the magnitude of adaptation for different levels of excitation, as reported by Kernell and Monster [59], and was assigned a value of 0.67. The parameter *d* was included to account for the observation that the absolute minimum firing rate that a MU can sustain is lower at derecruitment than at recruitment [18, 54, 118]. Such a lower firing rate at derecruitment may be partially due to adaptation. Therefore, firing rate could decay with time below the initially specified minimum firing rate by a small amount determined by *d*. In the present simulations, *d* was assigned a value of 2 imp/s, similar to values reported experimentally [54, 118].

As an example of how firing rate adaptation was implemented, consider one MU, say MU(60), in the population of n = 120 MUs. From Eq 2, the RTE for MU(60) = 6.96 excitation units. Under a constant excitatory drive of E = 20 excitation units, and in the absence of adaptation, Eq 3 would predict a steady firing rate of 21.04 imps/s. With adaptation, the adaptation current, A (Eq 12) progressively undercuts the net excitatory drive acting on the MU and decreases the firing rates. At 20 units of excitation, the maximum extent of firing rate adaptation (Eq 13) for MU60 would be *q(60)* = 0.67 [21.04–8 + 2] [(6.96–1)/(50–1)] = 1.23. After 15 s of activity, firing rate adaptation (Eq 12) for MU60 is = 1.23 x (1 –e^-15/22^) = 0.61 imp/s, and the adapted firing rate is 21.04–0.61 = 20.43 imp/s.

### Fatigue-related changes in motor unit force capacity

When rested and at maximum voluntary excitation (where E(t) = E_max_ = 67 excitation units), the modeled muscle generated a total maximum voluntary contraction force of 2,216 units and generated a minimum force of 1 force unit (0.045% MVC) at E(t) = 1. The model can be given a target at some percentage of the maximum force (e.g. 40% MVC would be 886.4 units of force). For the initial time sample (t = 0), the muscle was assumed to be completely rested and the model incremented excitation in 0.01 steps beginning with E(t) = 1. At each excitation step, the un-adapted firing rate (Eq 3), normalized firing rate (Eq 4), associated normalized force (Eqs 5 & 6), and the actual force developed (Eq 7) was determined for each MU. The total muscle force was calculated as the sum of force values across all MUs. If the total muscle force was below the target force, excitation was increased by 0.01. This process was repeated until the force target was met or slightly exceeded, at which time the model was advance 0.1 s (sample rate = 10 Hz) to the next time sample.

During each subsequent interval, the existing force capacity of each motor unit (P_E_) was calculated as P_E_ from the previous sample, minus the fatigue-related change during the 0.1 s interval (using Eq 10):

$${\text{P}}_{\text{E}}\left(\text{i},\text{t}\right)={\text{ P}}_{\text{E}}\left(\text{i},\text{t}-0.1\right)\;-\;0.10\left[\text{ FAT}\left(\text{i},\text{t}\right)\;\right]$$ (14)

Where P_E_(i,0) = P(i), as the muscle has not yet had time to fatigue. At each subsequent iteration and for each MU: (a) fatigue affected force capacity and contraction time, (b) the duration of activity (time since recruitment) affected firing rates, and (c) these factors affected normalized firing rates, normalized forces, and actual exerted forces. Thus, with sustained isotonic contractions, more excitation would be needed to meet the force target over the course of the contraction, possibly necessitating the recruitment of higher threshold MUs not initially active under rested conditions.

### Simulations

For target force levels at 15% MVC, and from 20–90% MVC in 10% increments, simulations were run for the duration necessary for the muscle force capacity to fall below the designated target force, and this duration was considered to be the endurance time. In addition to simulating total muscle force and endurance time, the model also enabled tracking of the instantaneous force and force capacity (absolute and relative) of each MU throughout the simulated contractions. A simulation was also run with a 100% MVC target for 200 s so it could be compared to the time-histories reported for this type of task in a number of experimental studies. Similarly, simulations were also performed using targets of 15%, 50% and 85% MVC, with each running until the total muscle force had decreased to 15% of maximum. This provided an interesting opportunity to compare the force capacities across MUs at the end of these trials for which the defined level of total muscle fatigue (85% decrease in total muscle force capacity) would be the same in all cases. In addition, we simulated fatigue involving a ‘staircase’ task in which force was maintained for 32 s at progressively increasing 20% MVC increments with a 5-s linear ramp between steps.

#### Comparison to experimental data

Endurance time data, compiled from the literature, were used to evaluate the validity of the fatigue model. Toward this goal, Frey Law & Avin (2010) provided an excellent summary of isometric fatigue collected over 194 studies in human subjects. We calculated mean endurance times by digitizing values from their Fig 4 for ankle (dorsiflexors: n = 20, plantarflexors: n = 12), knee (extensors: n = 99), trunk (flexors: n = 1, extensors: n = 12, rotators: n = 3), shoulder (flexors: n = 3, extensors: n = 3, abductors: n = 5), elbow (flexors: n = 79, extensors: n = 20) and hand tasks (grip: n = 37, digit: n = 7) at 15% (n = 9), 20% (n = 43), 30% (n = 40), 40% (n = 56), 50% (n = 53), 60% (n = 29), 70% (n = 19), 80% (n = 23) and 90% (n = 2) MVC. Weighted averages of endurance time were then calculated for each combination of %MVC and joint, and for each %MVC across joints, based on the number of means in each condition (from Table 1 of Frey Law & Avin, 2010). Maximum voluntary contractions are unique in that total muscle force capacity will start to decrease immediately (i.e. endurance time ≈ 0 s). Therefore, for 100% MVC contractions, force curves were digitized from Bigland-Ritchie et al. [36], Bigland-Ritchie [37], Kent-Braun et al. [38], and Jones et al. [39] so that polynomial regression equations could be established to approximate the time-history of force capacity decreases (r^2^ ≥ 0.99 for each curve). Those equations were used to calculate the average duration until a 1% MVC drop with a 100% MVC load.
